# Association between dietary intake of niacin and stroke in the US residents: evidence from national health and nutrition examination survey (NHANES) 1999–2018

**DOI:** 10.3389/fnut.2024.1391023

**Published:** 2024-07-19

**Authors:** Jie-Yu Qiu, Wen-Hui Zhang, Xiao-Ming Zhu, Li-Da Wu, Ji-Hua Huang, Jie Zhang

**Affiliations:** ^1^Department of Cardiology, XiShan People’s Hospital of Wuxi City, Wuxi, China; ^2^Department of Hepatobiliary Surgery, Beijing Chao-Yang Hospital Affiliated to Capital Medical University, Beijing, China; ^3^Department of Cardiology, Nanjing First Hospital, Nanjing Medical University, Nanjing, China; ^4^Guangdong Province Panyu Prison Hospital, Panyu, China

**Keywords:** niacin, stroke, NHANES, cross-sectional study, RCS

## Abstract

**Objective:**

This study aims to explore the association between niacin intake and stroke within a diverse, multi-ethnic population.

**Methods:**

A stringent set of inclusion and exclusion criteria led to the enrollment of 39,721 participants from the National Health and Nutrition Examination Survey (NHANES). Two interviews were conducted to recall dietary intake, and the USDA’s Food and Nutrient Database for Dietary Studies (FNDDS) was utilized to calculate niacin intake based on dietary recall results. Weighted multivariate logistic regression was employed to examine the correlation between niacin and stroke, with a simultaneous exploration of potential nonlinear relationships using restricted cubic spline (RCS) regression.

**Results:**

A comprehensive analysis of baseline data revealed that patients with stroke history had lower niacin intake levels. Both RCS analysis and multivariate logistic regression indicated a negative nonlinear association between niacin intake and stroke. The dose-response relationship exhibited a non-linear pattern within the range of dietary niacin intake. Prior to the inflection point (21.8 mg) in the non-linear correlation between niacin intake and stroke risk, there exists a marked decline in the risk of stroke as niacin intake increases. Following the inflection point, the deceleration in the decreasing trend of stroke risk with increasing niacin intake becomes evident. The inflection points exhibit variations across diverse populations.

**Conclusion:**

This investigation establishes a negative nonlinear association between niacin intake and stroke in the broader American population.

## Introduction

Niacin, also known as Vitamin B3, is a water-soluble vitamin with various benefits for human health (1). Niacin can be obtained through various foods such as fish, nuts, whole grains, and can also be supplemented through supplements. As widely known, niacin plays a positive role in maintaining skin health and helps prevent skin issues such as dermatitis and dryness (2). As an antioxidant, niacin helps combat damage caused by free radicals, thereby contributing to slowing down the aging process (3). Existing studies have shown that niacin participates in the energy metabolism process within the body, aiding in the conversion of food into usable energy, and is crucial for maintaining normal metabolism (4–6). Besides, niacin helps regulate cholesterol levels in the blood, particularly by lowering low-density lipoprotein cholesterol (LDL-C), promoting cardiovascular health (7). However, the association of dietary intake of niacin and the prevalence of stroke still remains unclear.

Globally, stroke is one of the major causes of death and disability (8–10). According to data from the World Health Organization (WHO), there are over 15,000,000 new cases of stroke worldwide each year. In developed countries, the incidence and mortality rates of stroke are relatively lower due to improved healthcare conditions and lifestyles (8, 11). However, in some developing countries, the incidence of stroke may be higher due to factors such as poor lifestyle choices, hypertension, diabetes, and other risk factors (12–14). Dyslipidemia is one of the important risk factors for stroke, as it can lead to the formation of atherosclerosis, narrowing the blood vessel walls and making them prone to thrombosis, thereby increasing the risk of stroke (15–17). Particularly, elevated LDL-C levels are considered a major driving factor for the development of atherosclerosis (18, 19). Numerous studies have demonstrated that Vitamin B3 has significant neuroprotective effects, including the enhancement of vascular function, reduction of oxidative stress, and improvement of lipid profiles. For instance, research by Cui et al. showed that Vitamin B3 supplementation reduced the incidence of ischemic strokes in animal models by promoting angiogenesis and neuronal survival (20). Additionally, clinical studies such as those by Teo et al. (21) have indicated that higher dietary intake of niacin is associated with a lower risk of stroke in human populations (21). These findings highlight the potential of niacin as a preventive measure against stroke, supporting its relevance in our study.

Due to its potential benefits in reducing LDL-C, increasing HDL-C levels, decreasing triglyceride levels, and improving overall lipid profile, niacin may lower the risk of stroke by reducing lipid metabolism abnormalities (20, 21). Niacin can also reduce oxidative stress levels, maintain endothelial function, and decrease vascular wall damage, thereby lowering the risk of atherosclerosis and thrombosis formation (22). These benefits contribute to maintaining vascular health, thus aiding in the prevention of stroke. Currently, there is insufficient scientific evidence to suggest that a significant reduction in the occurrence of stroke can be achieved by appropriately increasing niacin intake. Although niacin has shown regulatory effects on lipid metabolism and oxidative stress in some studies, its exact effectiveness in stroke prevention still requires further research for confirmation.

In the present study, we enrolled eligible participants from NHANES database and conducted a cross-sectional analysis to explore the relationship between dietary intake of niacin and the prevalence of stroke in a large normal population.

## Methods

### Study population

**FIGURE 1:**
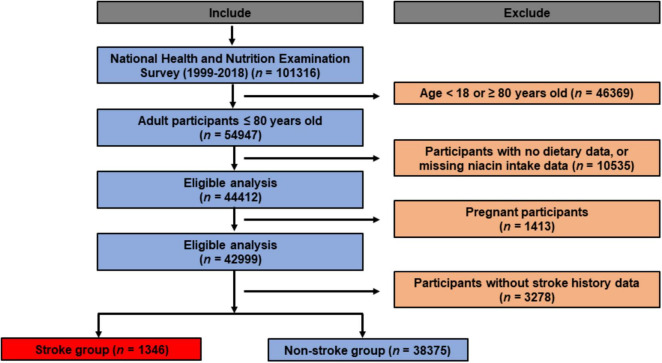
Participants enrollment flowchart.

NHANES is a nationally representative survey conducted throughout the United States, selecting participants through random sampling to ensure representative data for the entire U.S. population. It is implemented in two-year cycles. Each cycle involves face-to-face interviews, physical examinations, laboratory tests, and other health measurements conducted on thousands of residents (23). The data from NHANES are widely utilized in shaping public health policies, guiding research, and evaluating national health objectives. Researchers, policymakers, and the public have access to NHANES’ database to obtain information about the health and nutritional status of the U.S. population. We initially included 101,316 participants in the present study. Exclusion criteria as follows: (1) participants aged below 18 or above 80 years (n = 46,369); (2) participants with no dietary data, or missing niacin intake data (n = 10,535); (3) pregnant participants (n = 1,413); (4) individuals lacking stroke status (n = 3,278). Eventually, a total of 39721 participants were ultimately included. The flowchart of recruitment process can be found in Figure 1.

### Assessment of dietary intake of niacin

Dietary intake of niacin was obtained through interview, also known as What We Eat in America (WWEIA). The US Department of Agriculture (USDA) and the US Department of Health and Human Services (DHHS) collaboratively carried out the interview. All eligible NHANES participants undergo two 24-hour dietary recall interviews to disclose the types and amounts of foods they consumed in the 24 hours prior to the interview (from midnight to midnight). The initial dietary recall takes place in person at the Mobile Examination Center (MEC), and the subsequent recall is conducted via a phone interview around 3 to 10 days later. To calculate the nutrients and food components in various food items, the USDA’s Food and Nutrient Database for Dietary Studies (FNDDS) is utilized (24). The dataset, which encompasses the overall nutrient intakes, acts as a brief documentation of each individual’s nutrient intake. For this research, the participants’ daily niacin intake is established by computing the average of their two dietary recalls.

### Assessment of stroke

Stroke identification in this study relied on individuals disclosing a previous diagnosis from a medical practitioner during in-person interviews. Those who answered positively to the question, “Have you ever been told by a doctor or healthcare provider that you had a stroke?” were considered to have history of stroke. It’s essential to acknowledge that using self-reported data can be influenced by memory bias, potentially impacting how the information is interpreted (25). Furthermore, while the NHANES database lacks specific details about the stroke types, it’s reasonable to assume that a significant portion of participants identified as stroke cases likely had ischemic strokes.

### Covariates

Demographic data were obtained using standardized surveys that covered gender, race/ethnicity, educational background, smoking habits, and alcohol consumption. Alcohol consumption was defined as having consumed at least 12 drinks in the year before the survey. Body mass index (BMI) was utilized to assess overweight and obesity, with values exceeding 25 and 30 indicating overweight and obesity, respectively (26). Trained clinicians measured systolic/diastolic blood pressure (SBP/DBP), and the final blood pressure reading was calculated as the average of three consecutive readings taken at half-minute intervals. Laboratory tests, conducted following standardized procedures, determined various parameters. Participants meeting any of the following criteria were classified as having hypertension: (1) Average systolic blood pressure (SBP) ≥ 140 mmHg; (2) Average diastolic blood pressure (DBP) ≥ 90 mmHg; (3) Self-reported hypertension diagnosis; (4) Current use of antihypertensive medications (27). Individuals with a previous diagnosis of diabetes by a physician or health professional were categorized as having diagnosed diabetes (28–30).

### Statistical methods

Due to the complex sampling methods employed in the NHANES survey, our analytical approaches incorporated sample weights customized for specific research periods to ensure accurate calculations of health-related statistics. These weights adjust for the survey design, non-response, and post-stratification to make the results representative of the U.S. population. Weighted means and 95% confidence intervals were utilized to represent variables, ensuring that our estimates accurately reflect the population parameters. In examining variations in baseline traits between participants with and without stroke, continuous variables were analyzed using the student’s t-test, which assumes that the data are normally distributed and compares the means of two independent groups. For categorical variables, the chi-square test was employed, assessing the association between two categorical variables by comparing the observed frequencies to the expected frequencies under the null hypothesis of independence. Niacin intake was stratified into four quartiles to evaluate its relationship with stroke, with the lowest quartile (Q1) serving as the reference category. This stratification helps in understanding the dose-response relationship between niacin intake and stroke risk. Assessing the association of niacin with stroke involved employing multivariate logistic regression models. These models were adjusted for potential confounders such as age, sex, BMI, smoking status, physical activity, and other dietary factors. Odds ratios (ORs) and 95% confidence intervals (CIs) were calculated to estimate the strength of association between niacin intake and the likelihood of stroke. To explore the potential non-linear relationship between niacin intake and stroke, restricted cubic spline (RCS) regression with three knots (10th, 50th, and 90th percentiles) was utilized. RCS regression allows for flexibility in modeling non-linear associations by fitting smooth curves to the data without assuming a specific functional form. Subgroup analyses based on age, sex, and BMI were conducted to investigate whether the association between niacin intake and stroke varied across different population subgroups. Interaction terms were included in the regression models to test for statistical interaction, and stratified analyses were performed to provide subgroup-specific estimates. The statistical analyses were conducted using R software version 4.1.6 (http://www.R-project.org, The R Foundation, Vienna, Austria). All tests were two-tailed, with statistical significance set at a P-value < 0.05, ensuring that our findings are robust and reliable. Sensitivity analyses were also performed to check the stability of our results by using different model specifications and adjusting for additional potential confounders.

## Results

### Baseline characteristics

We firstly included 101,316 participants from NHANES 1999–2018, after applying the inclusion and exclusion criteria, 39,721 eligible participants were ultimately enrolled (Figure 1). The average age of the entire study cohort was 46.3 years, with almost half (48.6%) being male. A total of 1373 participants (3.5%) were assigned to stroke group. Notably, individuals with stroke history tended to be older (stroke vs. non stroke group: 60.4% vs. 45.9%) and had higher prevalence rates of hypertension (stroke vs. non stroke group: 77.4% vs. 35.7%) and diabetes (stroke vs. non stroke group: 35.5% vs. 11.8%). Detailed demographic and clinical characteristics are provided in Table 1, and Supplementary Table 1 presents a breakdown of these features based on niacin intake quantiles. The mean niacin intake for the overall study population was 21.99 mg, individuals with stroke history showing a lower mean intake of niacin (stroke vs. non stroke group: 21.5 vs. 25.6 mg). The stroke group exhibited elevated levels of HbA1c (stroke vs. non stroke group: 6.04% vs. 5.55%), fasting blood glucose (stroke vs non stroke group: 6.59 mmol/L vs. 5.81 mmol/L), fasting blood insulin (stroke vs. non stroke group: 103.93 pmol/L vs. 76.76 pmol/L), insulin resistance (stroke vs non stroke group: 5.83 vs. 3.57), triglycerides (stroke vs. non stroke group: 1.76 mmol/L vs. 1.49 mmol/L), and hypersensitive C-reactive protein (stroke vs. non stroke group: 0.65 mg/L vs. 0.4 mg/L). Additionally, participants with stroke history demonstrated a decreased level of high-density lipoprotein cholesterol (stroke vs. non stroke group: 1.31 mmol/L vs. 1.37 mmol/L), as outlined in Table 2.

**TABLE 1:** Baseline Characteristics Grouped by Stroke.

Variables	Overall (*n* = 39721)	Non- Stroke (*n* = 38375)	Stroke (*n* = 1346)	*P* value
Age, years				< 0.001***
18–40 years	38.94 [37.49, 40.39]	39.72 [38.71, 40.73]	8.46 [6.39, 10.52]	
40–60 years	39.94 [38.17, 41.70]	40.05 [39.22, 40.88]	35.32 [32.15, 38.50]	
> 60 years	21.12 [19.99, 22.26]	20.23 [19.49, 20.97]	56.22 [52.98, 59.45]	
Sex-male, %	48.55 [46.76, 50.35]	48.65 [48.14, 49.16]	44.71 [41.62, 47.79]	0.02*
Race, %				< 0.001***
Non-Hispanic White	69.29 [65.14, 73.44]	69.31 [67.31, 71.31]	68.41 [64.57, 72.24]	
Non-Hispanic Black	11.03 [10.10, 11.96]	10.90 [9.81, 11.99]	16.26 [13.96, 18.56]	
Mexican American	7.84 [6.93, 8.75]	7.91 [6.90, 8.92]	5.14 [3.86, 6.41]	
Other Hispanic	5.56 [4.67, 6.45]	5.61 [4.70, 6.52]	3.64 [2.25, 5.03]	
Other	6.28 [5.79, 6.77]	6.27 [5.75, 6.79]	6.56 [4.66, 8.45]	
Smoking, %	21.54 [20.45, 22.62]	21.36 [20.60, 22.12]	28.78 [25.64, 31.92]	< 0.001***
Drinking, %	83.74 [80.53, 86.96]	89.20 [88.31, 90.09]	84.94 [82.06, 87.81]	< 0.001***
Education level, %				< 0.001***
Below high school	5.08 [4.70, 5.46]	4.96 [4.58, 5.34]	9.97 [7.91, 12.02]	
High school	34.84 [33.07, 36.61]	34.55 [33.33, 35.76]	47.54 [44.02, 51.06]	
Above high school	60.00 [57.45, 62.55]	60.49 [59.13, 61.85]	42.50 [38.84, 46.15]	
SBP, mmHg	121.61 [121.27, 121.94]	121.39 [121.06, 121.73]	130.10 [128.54, 131.67]	< 0.001***
DBP, mmHg	71.66 [71.36, 71.95]	71.68 [71.38, 71.98]	70.70 [69.87, 71.54]	0.02*
DM, %	12.34 [11.72, 12.95]	11.75 [11.27, 12.22]	35.48 [31.99, 38.98]	< 0.001***
eGFR, ml/min/1.73m^2^	94.75 [94.26, 95.24]	95.20 [94.71, 95.69]	76.76 [75.04, 78.48]	< 0.001***
RBC, × 10^9^/L	4.72 [4.71, 4.74]	4.73 [4.72, 4.74]	4.59 [4.55, 4.64]	< 0.001***
WBC, × 10^9^/L	7.24 [7.20, 7.29]	7.23 [7.19, 7.28]	7.51 [7.36, 7.66]	< 0.001***
NE, × 10^9^/L	4.29 [4.26, 4.32]	4.28 [4.25, 4.32]	4.57 [4.46, 4.69]	< 0.001***
Monocyte, × 10^9^/L	0.56 [0.55, 0.56]	0.56 [0.55, 0.56]	0.59 [0.58, 0.61]	< 0.001***
LY, × 10^9^/L	2.14 [2.13, 2.16]	2.15 [2.13, 2.16]	2.07 [2.02, 2.12]	0.01*
PLT, × 10^6^/L	255.09 [253.69, 256.49]	255.28 [253.90, 256.66]	247.61 [241.09, 254.13]	0.02*
Hemoglobin, g/L	14.36 [14.32, 14.40]	14.37 [14.33, 14.41]	14.01 [13.88, 14.13]	< 0.001***
CHD, %	3.18 [2.89, 3.47]	2.80 [2.54, 3.05]	18.52 [15.81, 21.23]	< 0.001***
Angina, %	2.24 [2.00, 2.47]	1.97 [1.78, 2.17]	12.89 [10.33, 15.45]	< 0.001***
HF, %	2.01 [1.83, 2.19]	1.67 [1.51, 1.83]	15.73 [13.29, 18.17]	< 0.001***
Hypertension, %	36.69 [35.10, 38.29]	35.66 [34.78, 36.54]	77.36 [74.34, 80.38]	< 0.001***
Heart attack, %	3.17 [2.90, 3.44]	2.73 [2.52, 2.95]	20.54 [17.59, 23.48]	< 0.001***

Continuous variables are presented as the mean [95% CI], category variables are presented as the proportion [95% CI]. CI, confidence interval; SBP, systolic blood pressure; DBP, diastolic blood pressure; DM, diabetes; eGFR, estimated glomerular filtration rate; BMI, body mass index; WC, waist circumference; RBC, red blood cells; WBC, white blood cells; NE, neutrophils; LY, lymphocytes; PLT, platelets; CHD, coronary artery disease; HF, heart failure.

*P-value < 0.05, **P-value < 0.01, ***P-value < 0.001.

**TABLE 2:** Metabolic Indexes of the Study Population Grouped by Niacin Intake.

Variables	Q1 (0–15.81mg)	Q2 (15.81–21.99mg)	Q3 (21.99–29.85mg)	Q4 (29.85-72.63)w	*P* value
HbA1c, %	5.59 (5.56, 5.62)	5.60 (5.57, 5.62)	5.56 (5.54, 5.58)	5.52(5.50,5.54)	< 0.001***
FBG, mmol/L	5.81 (5.76, 5.86)	5.82 (5.75, 5.88)	5.85 (5.79, 5.90)	5.82(5.76,5.88)	< 0.001***
FBI, pmol/L	78.07 (75.07, 81.06)	77.24 (73.90, 80.58)	76.74 (73.36, 80.12)	77.89(74.76,81.01)	< 0.001***
HOMA-IR	3.72 (3.53, 3.90)	3.55 (3.37, 3.73)	3.65 (3.43, 3.87)	3.60(3.43,3.78)	< 0.001***
TG, mmol/L	1.46 (1.42, 1.50)	1.45 (1.40, 1.50)	1.54 (1.49, 1.59)	1.53(1.48,1.59)	< 0.001***
TC, mmol/L	5.18 (5.15, 5.21)	5.10 (5.07, 5.14)	5.09 (5.06, 5.13)	5.01(4.99,5.04)	< 0.001***
HDL-C, mmol/L	1.41 (1.40, 1.43)	1.40 (1.39, 1.41)	1.36 (1.35, 1.38)	1.31(1.30,1.32)	< 0.001***
LDL-C, mmol/L	3.08 (3.04, 3.11)	3.01 (2.97, 3.05)	3.01 (2.98, 3.05)	2.96(2.93,3.00)	< 0.001***
CRP, mg/L	0.48 (0.46, 0.50)	0.44 (0.41, 0.46)	0.39 (0.37, 0.41)	0.33(0.31,0.35)	< 0.001***

Continuous variables are presented as the mean [95% CI], category variables are presented as the proportion [95% CI]. CI, confidence interval; FBG, fasting blood glucose; FBI, fasting blood insulin; HbA1c, glycated hemoglobin; HDL-C, high-density lipoprotein cholesterol; CRP, C reactive protein; HOMA-IR, Homeostasis model assessment-insulin resistance; LDL-C, low-density lipoprotein cholesterol.

***P-value < 0.001.

### Associations of the intake of niacin with stroke

**FIGURE 2:**
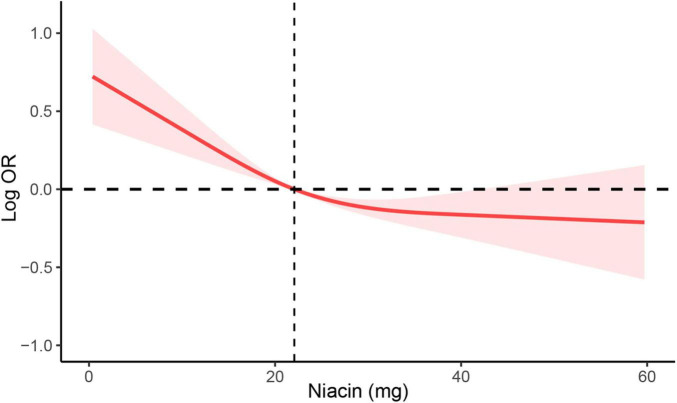
RCS analysis of the association between niacin intake and stroke. RCS analysis was adjusted for age, sex, race/ethnicity, education levels, smoking, drinking, hypertension, and DM. RCS, restricted cubic spline; DM, diabetes; OR, odds ratio.

To investigate the potential association between niacin consumption and stroke, we conducted a thorough multivariate analysis, considering variables like age, gender, ethnicity, education levels, smoking, drinking, hypertension, and diabetes. We found that niacin intake negatively associated with the risk of stroke before (OR: 0.97; 95% CI: 0.96-0.98) and after (OR: 0.98; 95% CI: 0.98-0.99) adjusting covariables. Moreover, participants were evenly divided into quartiles based on niacin intake, revealing that those with higher intake of niacin had the lower stroke risk before and after adjusting covariables (Table 3). Utilizing RCS analysis, we identified a negative nonlinear relationship between niacin intake and stroke risk (P for non-linear trend < 0.05). Before the inflection point (21.8 mg) of the non-linear relationship between niacin intake and the risk of stroke, there is a significant downward trend in the risk of stroke with increasing niacin intake. After the inflection point, the trend of decreasing stroke risk with increasing niacin intake slows down (Figure 2).

**TABLE 3:** Weighted Logistic Regression Analysis on the Association between Niacin and Stroke.

	Non-adjusted model		Model I		Model II	
	OR [95% CI]	*P* value	OR [95% CI]	*P* value	OR [95% CI]	*P* value
Continuous Niacin	0.97 [0.96, 0.98]	< 0.001***	0.98 [0.97, 0.99]	< 0.001***	0.98 [0.98, 0.99]	< 0.001***
Q1 (0–15.81mg)	Reference	–	Reference	–	Reference	–
Q2 (15.81–21.99mg)	0.78 [0.65, 0.93]	0.01*	0.81 [0.68, 0.97]	0.02*	0.88 [0.75, 1.05]	0.88
Q3 (21.99–29.85mg)	0.56 [0.46, 0.68]	< 0.001***	0.64 [0.52, 0.79]	< 0.001***	0.68 [0.55, 0.86]	< 0.001***
Q4 (29.85–72.63)	0.43 [0.35, 0.52]	< 0.001***	0.59 [0.47, 0.74]	< 0.001***	0.68 [0.53, 0.86]	< 0.001***

Data are presented as OR (95% CI). Model I adjusted for age, sex, and race/ethnicity. Model II adjusted for age, sex, race, education levels, smoking, drinking, hypertension, DM, and energy intake.

***P-value < 0.001, **P-value < 0.01, *P-value < 0.05.

### Subgroup analysis on the association of the intake of niacin and stroke

**FIGURE 3:**
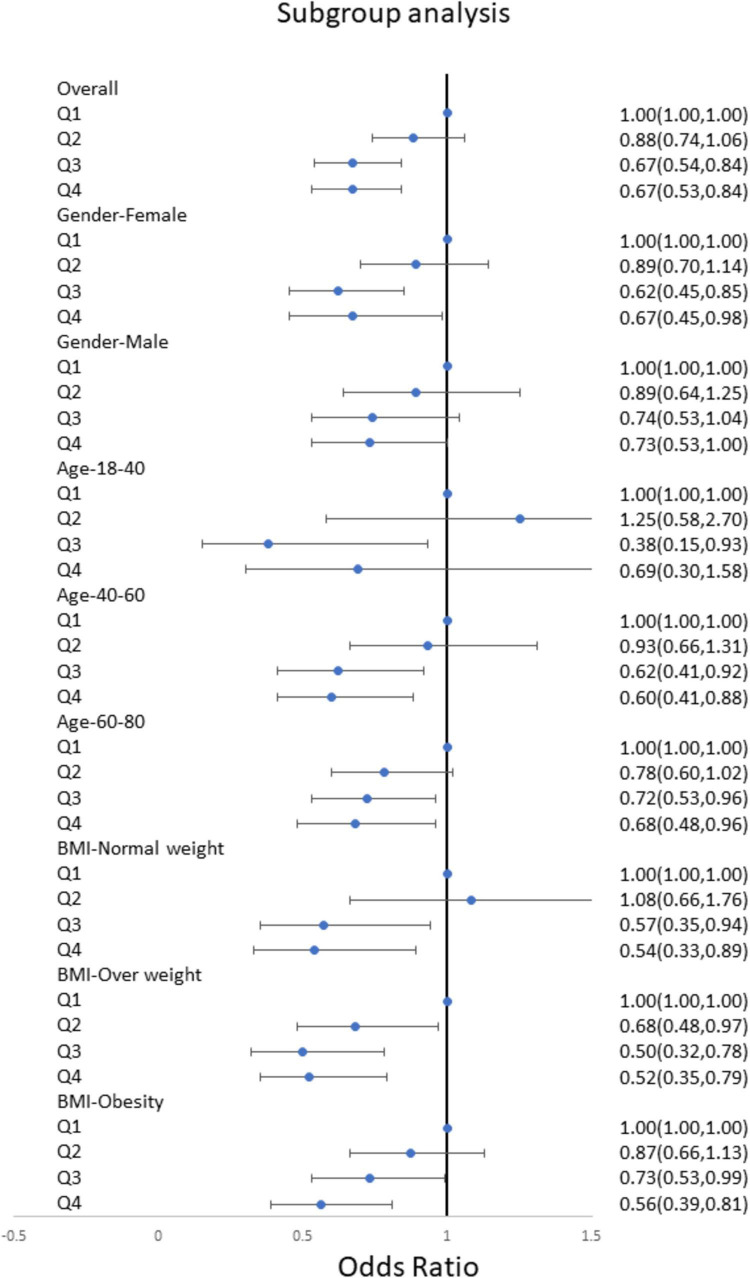
Subgroups multivariate logistic regression analyses for the association between niacin and stroke among different populations. Analyses were stratified by age, sex, BMI. Multivariate logistic regression analyses were adjusted for age, sex, race/ethnicity, education levels, smoking, drinking, hypertension, and DM. DM, diabetes; BMI, body mass index; OR, odds ratio.

**FIGURE 4:**
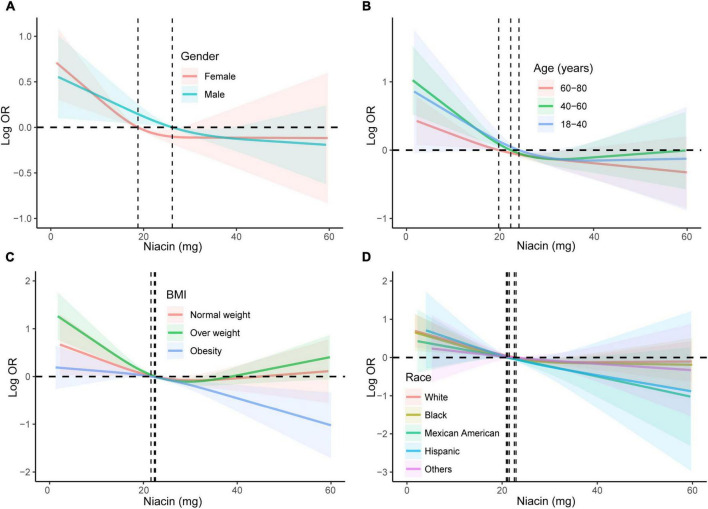
Subgroup analyses using RCS were conducted to examine the association between niacin and stroke across diverse demographic groups. The analyses were stratified based on gender **(A)**, age **(B)**, BMI **(C)**, and race/ethnicity **(D)**. Adjustment for potential confounders, including age, gender, race/ethnicity, education levels, smoking, drinking, hypertension, DM, and energy intake, was performed in the RCS analyses. RCS, restricted cubic spline; DM, diabetes; BMI, body mass index; OR, odds ratio.

We conducted subgroup analyses stratified by gender, age, and BMI to further verify the relationship between niacin intake and the risk of stroke in different populations. The results showed a significant downward trend in the risk of stroke with increasing niacin intake in various groups, including males, females, young, middle-aged, elderly, normal weight, overweight, and obese individuals (Figure 3). This confirms the stability of our findings across different demographic groups. Subgroup analyses of RCS were also conducted across diverse populations, revealing a negative nonlinear relationship between niacin intake and stroke in most groups (Figures 4A–D). Notably, the inflection points for this nonlinear association varied between males and females. Specifically, in females, the inflection point was identified at 19.2 mg, whereas in males, it occurred at 25.8 mg (Figure 4A). It is important to highlight that among overweight participants, the association between dietary niacin intake and stroke was U-shaped. After the inflection point, the stroke risk increased with the increase of niacin intake (Figure 4C).

### Sensitive analysis

There are some drawbacks of employing weighted analysis methods in the NHANES analysis. Weighted analysis is often utilized to account for sampling biases and ensure that the findings are reflective of the broader population. However, it’s important to consider that the weights are based on certain assumptions, and if these assumptions are not met, the results may be affected. One limitation is the reliance on self-reported data, which introduces the possibility of reporting errors or biases. Additionally, the weights are calculated based on specific demographic characteristics, and any changes or inaccuracies in these characteristics may impact the validity of the weighted analysis. Moreover, the effectiveness of the weighting method depends on the availability and accuracy of the data used for weight calculation. Furthermore, the use of weighted analysis assumes that the sampling design is adequately representative of the entire population. If there are limitations or shortcomings in the sampling approach, it may compromise the generalizability of the results. Therefore, in the present study, we also employed unweighted logistic regression to further confirm the conclusion. We found that the results of unweighted logistic regression were in accordance with main analysis using weighted logistic regression. Niacin intake negatively associated with the risk of stroke before (OR: 0.97; 95% CI: 0.96-0.97) and after (OR: 0.98; 95% CI: 0.97-0.98) adjusting covariables (Table 4).

**TABLE 4:** Unweighted Logistic Regression Analysis on the Association between Niacin and Stroke.

	Non-adjusted model		Model I		Model II	
	OR [95% CI]	*P* value	OR [95% CI]	*P* value	OR [95% CI]	*P* value
Continuous Niacin	0.97 [0.96, 0.97]	< 0.001***	0.98 [0.97, 0.98]	< 0.001***	0.98 [0.98, 0.99]	< 0.001***
Q1 (0–15.81mg)	Reference	–	Reference	–	Reference	-
Q2 (15.81–21.99mg)	0.75 [0.65, 0.86]	0.01*	0.80 [0.70, 0.92]	0.02*	0.86 [0.73, 1.00]	0.07
Q3 (21.99–29.85mg)	0.58 [0.50, 0.68]	< 0.001***	0.68 [0.59, 0.79]	< 0.001***	0.70 [0.6, 0.83]	< 0.001***
Q4 (29.85–72.63)	0.41 [0.35, 0.48]	< 0.001***	0.58 [0.49, 0.69]	< 0.001***	0.63 [0.52, 0.79]	< 0.001***

Data are presented as OR (95% CI). Model I adjusted for age, sex, and race/ethnicity. Model II adjusted for age, sex, race, education levels, smoking, drinking, hypertension, DM, and energy intake.

***P-value < 0.001, **P-value < 0.01, *P-value < 0.05.

## Discussion

In the present study, we conducted a cross-sectional analysis to explore the association of dietary niacin intake and stroke risk. We found that there was a negative nonlinear relationship between niacin and stroke. Prior to the inflection point (21.8 mg) in the non-linear correlation between niacin consumption and stroke risk, there is a notable decline in the likelihood of stroke as niacin intake increases. Subsequent to this inflection point, the decrease in stroke risk associated with higher niacin intake exhibits a decelerated trend.

Niacin, also known as vitamin B3, is a water-soluble vitamin essential for various physiological functions in the human body (31). Niacin is found in various foods, including meat, fish, nuts, and grains, and it can also be synthesized by the body from the amino acid tryptophan. Adequate niacin intake is important for maintaining overall health and preventing niacin deficiency, which can lead to a condition known as pellagra (32, 33). Additionally, niacin is sometimes used in higher doses for therapeutic purposes, such as managing certain lipid disorders (34, 35). Actually, previous studies have indicated that niacin plays important therapeutic roles in various diseases. Tian et al. revealed a negative relationship between dietary niacin intake and depression risk. Compared to the lowest niacin intake group (Q1, ≤ 15.96 mg/day), the adjusted odds ratios (OR) for depression in the higher intake groups (Q2, Q3, and Q4) showed a decreasing trend, with the lowest risk observed in Q4 (≥ 32.29 mg/day). The relationship remained consistent across different demographic subgroups, including sex, age, and BMI (36). Results from Xiang et al. (37) indicated a positive correlation between higher dietary niacin intake and several favorable outcomes. Increased niacin intake was associated with higher grip strength, total lean mass, appendicular lean mass, and total bone mineral content. Conversely, higher niacin intake showed a negative association with total fat, trunk fat, and sarcopenia risk. Notably, dietary niacin supplementation also demonstrated a significant reduction in homeostasis model assessment of insulin resistance (HOMA-IR), fasting blood glucose (in participants without diabetes), and fasting insulin (37). Lee et al. (38) also conducted a cross-section analysis and found that higher levels of niacin intake were associated with decreased odds of glaucoma overall and in women (38). However, to the best of our knowledge, the association of niacin intake and stroke risk remains exclusive.

Risk factors for stroke include high blood pressure, smoking, diabetes, obesity, and a sedentary lifestyle. Age, family history, and certain medical conditions also contribute to the risk (39, 40). Prevention measures often involve lifestyle changes such as maintaining a healthy diet, exercising regularly, managing blood pressure, and avoiding smoking and excessive alcohol consumption (41, 42). Many researchers have studied the risk factors for stroke in the NHANES database. There is an interesting study focused on the association of urinary paraxanthine levels and stroke risk. Authors found that there was a negative correlation between urinary paraxanthine levels and stroke risk. However, the negative association of urinary caffeine levels with stroke incidence was observed specifically in Mexican Americans, with no evident correlation in other populations, implying potential predictive and diagnostic implications in clinical practice (43). Another study also based on NHANES database also indicated a U-shaped correlation exists between serum uric acid levels and the risk of stroke. Both low and high SUA levels elevate the risk of stroke in distinct populations, with the exception being the other Hispanic population. Effective early management of SUA is crucial for preventing strokes in high-risk populations (44). Zhao et al. (45) utilized a larger sample size drawing on data from the NHANES spanning 2011 to 2018 to explore the association of blood selenium levels and the risk of stroke. With 13,755 adults aged 20 years and above, multivariate logistic regression models and dose-response analyses were employed. In the fully adjusted model, the highest tertile of blood selenium levels was also negatively associated with stroke compared to the lowest tertile (OR = 0.70, 95% CI: 0.53-0.93, P for trend = 0.016) (45). In the present study, we also utilized a large sample size from NHANES database to explore the relationship between niacin intake and stroke risk. We found that increased niacin intake may have protective effect on prevention of stroke. Especially prior of the inflection point of 21.8 mg, the stroke risk decreased significantly with the increase of niacin intake. It is worth noting that the inflection points vary among different populations. For instance, the inflection point for males is 25.8 mg, and for females, it is 19.2 mg. This variability may provide insights for the clinical application of niacin.

Currently, the exact mechanism of Niacin in preventing strokes is not fully understood, but Niacin may prevent the occurrence of strokes through the following mechanisms. Firstly, Niacin has a lipid-regulating effect, especially in lowering low-density lipoprotein cholesterol (LDL-C) levels and increasing high-density lipoprotein cholesterol (HDL-C) levels (21). By reducing lipid deposition on arterial walls, Niacin may help prevent atherosclerosis, thereby reducing the risk of strokes. Niacin possesses antioxidant properties, aiding in neutralizing free radicals and alleviating oxidative stress on blood vessels (46–48). This may contribute to maintaining vascular health and reducing the risk of strokes. Niacin is believed to have anti-inflammatory effects, mitigating inflammatory responses (49–51). As inflammation is associated with atherosclerosis and stroke occurrence, the anti-inflammatory effects of Niacin may contribute to stroke prevention. Finally, Niacin may have a vasodilatory effect by promoting the production of nitric oxide, contributing to the maintenance of vascular elasticity and function, ultimately reducing the risk of strokes (52, 53). However, these protective mechanisms are relatively superficial, and a more in-depth exploration of molecular biological mechanisms requires further investigation through animal experiments. The specific protective effects of niacin also need validation through large-scale prospective clinical trials.

Our study has several limitations that should be acknowledged and considered in future research: (1) The cross-sectional design inherently hinders establishing causality between niacin and stroke (54); (2) Using a single 24-hour recall may not be the optimal method for calculating habitual dietary intake at an individual level due to day-to-day variations. The large number of participants in NHANES surveys may limit the practical application of more accurate options [e.g., multiple 24-hour recalls, food frequency questionnaire (FFQ)] in exploring the long-term link between dietary intake and hypertension. This issue should be addressed in future studies utilizing NHANES dietary information (55); moreover, tryptophan, an essential amino acid, can be converted into niacin through metabolic pathways, thereby influencing overall niacin levels. The absence of data on tryptophan in the study’s database prevents a comprehensive analysis of niacin metabolism and its dietary implications. Future research should consider incorporating measures of tryptophan alongside niacin intake to provide a more accurate assessment of their interplay and nutritional impact; (3) Despite attempts to include numerous covariates to control confounding bias, stroke is a complex disorder influenced by multiple genetic, behavioral, and environmental factors. Unidentified confounders may exist, affecting the pathogenesis and progression of stroke, as not all relevant factors are explicitly documented in the NHANES database; Self-reporting is a convenient means of obtaining information about dietary intake and hypertension occurrence among NHANES participants. However, this method may introduce recall bias, and caution is warranted during the analysis and interpretation of the data.

## Conclusion

We utilized a substantial sample size from the NHANES database to examine the association between niacin intake and the risk of stroke. Our findings suggest that heightened niacin intake may confer a protective effect in preventing strokes. There is a negative nonlinear association of niacin intake and stroke. Notably, the inflection points exhibit variations across diverse populations. This diversity in inflection points could offer valuable insights for the clinical utilization of niacin.

## Data availability statement

The original contributions presented in the study are included in the article/Supplementary material, further inquiries can be directed to the corresponding author.

## Ethics statement

The studies involving humans were approved by The NCHS Ethics Review Board protects the rights and welfare of NHANES participants. The NHANES protocol complies with the U.S. Department of Health and Human Services Policy for the Protection of Human Research Subjects. NCHS IRB/ERC Protocol number: 2011-17. Ethical review and approval were waived for this study as it solely used publicly available data for research and publication. The studies were conducted in accordance with the local legislation and institutional requirements. The participants provided their written informed consent to participate in this study.

## Author contributions

J-YQ: Formal analysis, Investigation, Methodology, Resources, Validation, Writing−original draft. W-HZ: Conceptualization, Data curation, Validation, Writing−original draft. X-MZ: Data curation, Formal analysis, Writing−original draft. L-DW: Conceptualization, Supervision, Writing−original draft, Writing−review and editing. J-HH: Conceptualization, Data curation, Writing−review and editing. JZ: Conceptualization, Writing−original draft, Writing−review and editing.
