# Supplementary material for: Association between dietary intake of niacin and stroke in the US residents: evidence from national health and nutrition examination survey (NHANES) 1999–2018
Source: Front Nutr. 2024 Jul 19;11:1391023. doi: 10.3389/fnut.2024.1391023 (PMC11294223; doi:10.3389/fnut.2024.1391023)
Supplement: Supplementary file 1 [file Table_1.docx]

**Table S1. Clinical Characteristics of the Study Population Grouped by CDAI quantiles.**

| Variables | CDAI-Q1 | CDAI-Q2 | CDAI-Q3 | CDAI-Q4 | *P* value |
| --- | --- | --- | --- | --- | --- |
| Age, years |  |  |  |  | <0.001*** |
| 18-40 years | 35.01 [33.68, 36.34] | 34.89 [33.51, 36.28] | 38.58 [37.00, 40.16] | 45.85 [44.25, 47.44] |  |
| 40-60 years | 38.46 [37.23, 39.69] | 40.43 [39.15, 41.71] | 40.61 [39.28, 41.93] | 40.04 [38.60, 41.47] |  |
| > 60 years | 26.53 [25.42, 27.64] | 24.68 [23.47, 25.88] | 20.81 [19.63, 21.99] | 14.12 [13.13, 15.10] |  |
| Sex-male, % | 24.54 [23.48, 25.60] | 35.85 [34.67, 37.03] | 52.39 [51.18, 53.61] | 74.67 [73.74, 75.60] | <0.001*** |
| Race, % |  |  |  |  | <0.001*** |
| Non-Hispanic White | 64.47 [62.00, 66.93] | 68.67 [66.41, 70.92] | 71.02 [68.88, 73.16] | 71.96 [69.99, 73.92] |  |
| Non-Hispanic Black | 13.71 [12.19, 15.23] | 11.48 [10.20, 12.75] | 9.71 [8.69, 10.74] | 9.80 [8.73, 10.86] |  |
| Mexican American | 8.46 [7.26, 9.65] | 7.88 [6.79, 8.96] | 7.44 [6.35, 8.53] | 7.70 [6.68, 8.73] |  |
| Other Hispanic | 7.04 [5.46, 8.63] | 5.31 [4.41, 6.20] | 5.33 [4.42, 6.25] | 4.84 [4.02, 5.66] |  |
| Other | 6.32 [5.57, 7.07] | 6.67 [5.95, 7.40] | 6.50 [5.83, 7.17] | 5.70 [5.09, 6.32] |  |
| Smoking, % | 24.28 [23.11, 25.46] | 20.28 [19.01, 21.56] | 19.65 [18.53, 20.78] | 22.29 [21.18, 23.40] | <0.001*** |
| Drinking, % | 83.58 [82.16, 85.01] | 87.69 [86.62, 88.75] | 90.73 [89.68, 91.78] | 93.04 [92.14, 93.94] | <0.001*** |
| Education level, % |  |  |  |  | <0.001*** |
| Below high school | 7.93 [7.17, 8.68] | 5.54 [4.96, 6.11] | 4.34 [3.86, 4.83] | 3.17 [2.79, 3.56] |  |
| High school | 39.89 [38.31, 41.46] | 35.53 [33.85, 37.21] | 32.37 [30.86, 33.87] | 32.73 [31.09, 34.37] |  |
| Above high school | 52.19 [50.56, 53.82] | 58.93 [57.11, 60.75] | 63.29 [61.64, 64.94] | 64.09 [62.35, 65.84] |  |
| SBP, mmHg | 122.59 [122.03, 123.16] | 121.60 [121.07, 122.13] | 121.21 [120.75, 121.68] | 121.22 [120.79, 121.65] | <0.001*** |
| DBP, mmHg | 71.34 [70.94, 71.74] | 71.00 [70.60, 71.40] | 71.63 [71.22, 72.04] | 72.49 [72.14, 72.83] | <0.001*** |
| DM, % | 13.71 [12.88, 14.53] | 13.67 [12.72, 14.61] | 12.04 [11.32, 12.77] | 10.38 [9.77, 11.00] | <0.001*** |
| FBG, mmol/L | 5.81 [5.76, 5.86] | 5.82 [5.75, 5.88] | 5.85 [5.79, 5.90] | 5.82 [5.76, 5.88] | <0.001*** |
| HbA1c, % | 5.59 [5.56, 5.62] | 5.60 [5.57, 5.62] | 5.56 [5.54, 5.58] | 5.52 [5.50, 5.54] | <0.001*** |
| eGFR, ml/min/1.73m^2^ | 93.07 [92.39, 93.74] | 93.91 [93.19, 94.63] | 94.91 [94.28, 95.53] | 96.59 [95.99, 97.20] | <0.001*** |
| TG, mmol/L | 1.46 [1.42, 1.50] | 1.45 [1.40, 1.50] | 1.54 [1.49, 1.59] | 1.53 [1.48, 1.59] | <0.001*** |
| TC, mmol/L | 5.18 [5.15, 5.21] | 5.10 [5.07, 5.14] | 5.09 [5.06, 5.13] | 5.01 [4.99, 5.04] | <0.001*** |
| LDL-C, mmol/L | 1.41 [1.40, 1.43] | 1.40 [1.39, 1.41] | 1.36 [1.35, 1.38] | 1.31 [1.30, 1.32] | <0.001*** |
| HDL-C, mmol/L | 3.08 [3.04, 3.11] | 3.01 [2.97, 3.05] | 3.01 [2.98, 3.05] | 2.96 [2.93, 3.00] | <0.001*** |
| RBC, ×10^9^/L | 4.60 [4.59, 4.62] | 4.65 [4.63, 4.66] | 4.75 [4.73, 4.76] | 4.87 [4.85, 4.88] | <0.001*** |
| WBC, ×10^9^/L | 7.32 [7.24, 7.39] | 7.25 [7.18, 7.32] | 7.23 [7.17, 7.30] | 7.18 [7.12, 7.24] | 0.03* |
| NE, ×10^9^/L | 4.33 [4.27, 4.39] | 4.31 [4.25, 4.36] | 4.29 [4.24, 4.33] | 4.24 [4.20, 4.29] | 0.04* |
| Monocyte, ×10^9^/L | 0.55 [0.54, 0.56] | 0.55 [0.54, 0.56] | 0.56 [0.55, 0.57] | 0.57 [0.57, 0.58] | <0.001*** |
| LY, ×10^9^/L | 2.19 [2.17, 2.21] | 2.14 [2.12, 2.17] | 2.14 [2.11, 2.17] | 2.11 [2.09, 2.14] | <0.001*** |
| PLT, ×10^6^/L | 265.11 [262.92, 267.31] | 258.17 [256.09, 260.25] | 252.55 [250.73, 254.37] | 247.11 [245.18, 249.03] | <0.001*** |
| Hemoglobin, g/L | 13.92 [13.86, 13.97] | 14.11 [14.05, 14.16] | 14.43 [14.38, 14.48] | 14.86 [14.81, 14.91] | <0.001*** |
| CHD, % | 3.75 [3.29, 4.20] | 3.46 [2.99, 3.94] | 3.11 [2.66, 3.56] | 2.58 [2.18, 2.99] | 0.001** |
| Angina, % | 2.93 [2.42, 3.44] | 2.33 [1.98, 2.69] | 2.09 [1.74, 2.45] | 1.75 [1.44, 2.06] | <0.001*** |
| HF, % | 2.78 [2.36, 3.19] | 2.44 [2.10, 2.78] | 1.69 [1.42, 1.96] | 1.36 [1.06, 1.66] | <0.001*** |
| Hypertension, % | 40.56 [39.12, 41.99] | 37.58 [36.15, 39.02] | 36.35 [35.02, 37.69] | 33.22 [31.89, 34.54] | <0.001*** |
| Heart attack, % | 3.93 [3.45, 4.41] | 3.39 [2.93, 3.84] | 2.78 [2.38, 3.18] | 2.77 [2.35, 3.19] | <0.001*** |

Continuous variables are presented as the mean [95% CI], category variables are presented as the proportion [95% CI]. CI, confidence interval; SBP, systolic blood pressure; DBP, diastolic blood pressure; DM, diabetes; FBG, fasting blood glucose; HbA1c, glycated hemoglobin; eGFR, estimated glomerular filtration rate; BMI, body mass index; WC, waist circumference; TG, triglycerides; TC, total cholesterol; LDL-C, low-density lipoprotein cholesterol; HDL-C, high-density lipoprotein cholesterol; RBC, red blood cells; WBC, white blood cells; NE, neutrophils; LY, lymphocytes; PLT, platelets; CHD, coronary artery disease; HF, heart failure; . * *P* value<0.05, ** *P* value<0.01, *** *P* value<0.001.
